# Genome-Wide Identification of Candidate Genes for Milk Production Traits in Korean Holstein Cattle

**DOI:** 10.3390/ani11051392

**Published:** 2021-05-13

**Authors:** Sangwook Kim, Byeonghwi Lim, Joohyeon Cho, Seokhyun Lee, Chang-Gwon Dang, Jung-Hwan Jeon, Jun-Mo Kim, Jungjae Lee

**Affiliations:** 1 Department of Animal Science and Technology, Chung-Ang University, Anseong 17546, Gyeonggi-do, Korea; genemap4077@cau.ac.kr (S.K.); hwi1208@cau.ac.kr (B.L.); 2 Dairy Cattle Genetic Improvement Center, Nonghyup, Goyang 10292, Gyeonggi-do, Korea; dciccho@hanmail.net (J.C.); asdko123@nate.com (S.L.); 3 Animal Genetics and Breeding Division, National Institute of Animal Science, RDA, Cheonan 31000, Chungcheongnam-do, Korea; gkgkgki@korea.kr; 4 Animal Welfare Research Team, National Institute of Animal Science, RDA, Wanju 55365, Jeollabuk-do, Korea; jeon75@korea.kr

**Keywords:** milk production, Holstein cattle, genome-wide association studies, fine mapping, genomic selection

## Abstract

**Simple Summary:**

Milk production traits that are economically important in the dairy industry have been considered the main selection criteria for breeding. The present genome-wide association study was performed to identify chromosomal loci and candidate genes with potential effects on milk production phenotypes in a Korean Holstein population. A total of eight significant quantitative trait locus regions were identified for milk yield (*Bos taurus* autosome (BTA) 7 and 14), adjusted 305-d fat yield (BTA 3, 5, and 14), adjusted 305-d protein yield (BTA 8), and somatic cell score (BTA 8 and 23) of milk production traits. Furthermore, we discovered three main candidate genes (diacylglycerol O-acyltransferase 1 (*DGAT1*), phosphodiesterase 4B (*PDE4B*), and anoctamin 2 (*ANO2*)) through bioinformatics analysis. These genes may help to understand better the underlying genetic and molecular mechanisms for milk production phenotypes in the Korean Holstein population.

**Abstract:**

We performed a genome-wide association study and fine mapping using two methods (single marker regression: frequentist approach and Bayesian C (BayesC): fitting selected single nucleotide polymorphisms (SNPs) in a Bayesian framework) through three high-density SNP chip platforms to analyze milk production phenotypes in Korean Holstein cattle (*n* = 2780). We identified four significant SNPs for each phenotype in the single marker regression model: AX-311625843 and AX-115099068 on *Bos taurus* autosome (BTA) 14 for milk yield (MY) and adjusted 305-d fat yield (FY), respectively, AX-428357234 on BTA 18 for adjusted 305-d protein yield (PY), and AX-185120896 on BTA 5 for somatic cell score (SCS). Using the BayesC model, we discovered significant 1-Mb window regions that harbored over 0.5% of the additive genetic variance effects for four milk production phenotypes. The concordant significant SNPs and 1-Mb window regions were characterized into quantitative trait loci (QTL). Among the QTL regions, we focused on a well-known gene (diacylglycerol O-acyltransferase 1 (*DGAT1*)) and newly identified genes (phosphodiesterase 4B (*PDE4B*), and anoctamin 2 (*ANO2*)) for MY and FY, and observed that *DGAT1* is involved in glycerolipid metabolism, fat digestion and absorption, metabolic pathways, and retinol metabolism, and *PDE4B* is involved in cAMP signaling. Our findings suggest that the candidate genes in QTL are strongly related to physiological mechanisms related to the fat production and consequent total MY in Korean Holstein cattle.

## 1. Introduction

Milk production is an economically important trait affecting profitability in Holstein dairy cattle. Holstein selection for genetic improvement with economic characteristics in the past was based on a traditional best linear unbiased prediction together with an estimated breeding value (EBV), a traditional animal breeding program that depends only on phenotypic and pedigree information [1]. Recent advances in DNA-based marker technology have allowed us to identify quantitative trait loci (QTL), which are genomic regions related to complex characteristics such as milk yield in dairy cows, and it was reported that integrating detected QTL into genetic evaluations would promote the genetic improvement of productivity by providing a means to increase the accuracy of an estimation of an individual’s genetic ability.

For dairy cattle, since Georges et al. [2] reported a QTL mapping study on milk yield characteristics, more than 1350 QTL for traits related to milk production were reported (https://www.animalgenome.org/cgi-bin/QTLdb/BT/index accessed on 1 October 2020). The specific genes or mechanisms involved in the detection of QTL, however, have not been sufficiently characterized because of the low resolution of the traditional positional cloning approach using microsatellite markers [3] and the lack of whole-genome sequences. In addition, the limitations of QTL mapping using linkage analysis or linkage disequilibrium (LD) based on panels of low- to moderate-density markers are well documented [4].

Advances in DNA analysis technology led to the emergence of genome-wide panels containing hundreds of thousands of single nucleotide polymorphisms (SNPs), which have been widely used for detecting QTL for complex traits in many species [5], and its usefulness for detecting mutations has been demonstrated. Genome-wide association studies (GWAS) that use this high-density SNP chip technology were used to locate genes related to complex traits and have advantages in detecting effective causal alterations and defining narrow genomic regions containing causal alterations compared with the traditional QTL mapping strategies [6]. These techniques make it possible to detect SNPs and candidate gene markers related to the economic traits of dairy cattle accurately.

Previous GWAS were performed using various SNP chip platforms in cattle breeds. Two SNPs related to milk yield on *Bos taurus* autosome 4 (BTA 4), two SNPs related to fat yield on BTA 14/2, and one SNP related to fat percentage on BTA 1 were identified using the Bovine SNP50K BeadChip platform (Illumina, San Diego, CA, USA) in the Braunvieh cattle breed [7]. Dozens of SNPs related to milk yield or fat yield were found on BTA 14 (1.2–2.8 Mb) in Italian Holstein cattle, also through GWAS [8]. In addition, GWAS performed using the BovineHD SNP777K BeadChip in Canadian Holstein cattle for milk production, fat production, and protein production traits found many candidate SNPs and chromosomal regions [9], and many other candidate genes related to milk production, such as diacylglycerol O-acyltransferase 1 (*DGAT1*), transcriptional repressor GATA binding 1 (*TRPS1*), stearoyl-CoA desaturase (*SCD*), and growth hormone receptor (*GHR*) were found in GWAS using a high-density SNP chip platform [10,11,12,13,14,15]. GWAS of Korean Holstein were also performed using the Bovine SNP50K BeadChip platform (Illumina), indicating significant genetic regions such as *DGAT1* related to milk production, fat, and protein [16,17].

The ultimate goal of GWAS is to elucidate the causative alteration rather than the locus, and high-density SNPs are required to identify such causative alterations closely. Furthermore, higher-density fine mapping was performed recently using a customized SNP array in which SNPs were included in the existing commercialized SNP chip platform through whole-genome sequencing methods or other genotyping techniques [18,19,20]. Although causal alterations are not always directly identified by this approach, this GWAS fine mapping method can highlight a narrower genomic region as potentially containing the causal alteration [21,22].

Bioinformatics analysis provides useful information for understanding the biological and biochemical mechanisms of genes at the molecular level [23]. Recent studies reported highly reliable results by analyzing the pathway or protein-protein interaction (PPI) based on candidate genes associated with milk production in dairy cattle [24,25,26,27,28]. The aim of the present study was to identify chromosome regions and candidate genes related to four milk production traits—adjusted 305-day milk yield (MY), adjusted 305-day fat yield (FY), adjusted 305-day protein yield (PY), and somatic cell score (SCS)—in a Korean Holstein population using high-density SNP chip platforms and two models to perform GWAS and fine mapping.

## 2. Materials and Methods

### 2.1. Animals and Phenotypes

The multi-lactation single-trait model considering parity, provided by the National Institute of Animal Science, was applied to MY, FY, PY, and SCS traits. The estimates for genetic, residual variances and heritability for each trait are shown in Table S1. The deregressed EBV (DEBVs), as response variables used in this study, were re-estimated using the EBVs and the reliabilities of each individual. In addition, to explain the heterogeneous variance resulting from the different reliabilities for each individual from the re-estimated response variables, the weighting factor was calculated using the following formula [29] and was applied to GWAS:

(1) $$\omega_{i}=\frac{\left(1-h^{2}\right)}{c+\left[\frac{1-r_{i}^{2}}{r_{i}^{2}}\right]h^{2}}$$

where $r_{i}^{2}$ is the reliability of EBVs; $h^{2}$ is the heritability estimated for each trait; and *c* is the genetic variance ratio explained by the SNP marker information (assumed as 0.4) [30]. After converting response variables into DEBVs and excluding individuals with reliability (reliability EBVs—reliability of parent average) of 0.1 or less, the analysis of model for GWAS was performed with a group of 2780 animals (926 bulls and 1854 cows) whose genotype and phenotype data were available. The distributions of reliabilities are shown in Figure S1.

### 2.2. Genotyping

DNA samples (200 ng adjusted to 50 ng/µL) from 2780 Korean Holsteins (926 bulls and 1854 cows) were prepared from sampled hair according to standard protocols. DNA concentration and quality were measured using a NanoDrop ND-1000 spectrophotometer (NanoDrop Technologies, Wilmington, DE, USA). Genotypic data were obtained using three SNP panels: Illumina Bovine SNP50K v2 (*n* = 1095), Illumina Bovine SNP50K v3 (*n* = 1013), and Affymetrix Axiom Bovine Custom300K (*n* = 672) (Table S2).

Genome-wide SNP genotyping based on Ensembl *Bos taurus* UMD3.1 (http://oct2018.archive.ensembl.org/Bos_taurus/Info/Index accessed on 1 October 2020) was performed using either the Axiom Bovine Custom SNP300K array (Affymetrix Inc., Santa Clara, CA, USA) containing 297,424 SNPs or the Illumina Bovine SNP50K v2 and v3 BeadChip (Illumina, San Diego, CA, USA) containing 53,218 and 54,609 SNPs, respectively. To construct the customized Axiom array, SNPs were collected from previous commercial SNP genotyping platforms (i.e., Illumina Bovine50K versions and Affymetrix Bovine Genotyping array) and ranges of QTL regions (*DGAT1*, melanocortin 1 receptor (*MC1R*), EvC ciliary complex subunit 2 (*EVC2*), annexin A10 (*ANXA10*), GDNF family receptor alpha 2 (*GFRA2*), neurotrophin 3 (*NTF3*), argininosuccinate synthase 1 (*ASS1*), LDL receptor related protein 4 (*LRP4*), integrin subunit beta 2 (*ITGB2*), uridine monophosphate synthetase (*UMPS*), COPI coat complex subunit alpha (*COPA*), coagulation factor XI (*F11*), enoyl-CoA hydratase, short chain 1 (*ECHS1*), DNA polymerase lambda (*POLL*), myostatin (*MSTN*), zinc finger imprinted 2 (*ZIM2*), and anoctamin 2 (*ANO2*)) associated with various phenotypes in cattle including Holstein. Affymetrix Axiom Bovine Custom SNP300K Information of total SNPs in Affymetrix Axiom Bovine Custom SNP300K is described in Table S3.

### 2.3. Quality Control (QC) and Imputation

QC was performed with the SNPs and animals in each genotyping platform. First, for SNPs, the unmapped SNPs and sex chromosome SNPs were excluded, and then SNPs with a call rate < 0.95, minor allele frequency (MAF) < 0.01, and Hardy–Weinberg equilibrium (HWE) with *p*-value < 0.0001 were removed. Secondly, 57 individuals did not pass the call rate < 0.95 criterion, and additionally, 109 individuals whose genomic information differed from their pedigree information were also excluded based on a paternity test. After that, the imputation was performed based on the Affymetrix Axiom Bovine Custom SNP300K using FImputeV3 [31]. Finally, 2614 individuals with 201,704 SNPs were used for further analyses. Multidimensional scaling (MDS) was performed based on SNPs to indicate similarities between individuals.

### 2.4. GWAS

#### 2.4.1. Single Marker Regression (SMR)

PLINK 1.9 [32] was used to perform an association analysis between SNP markers and DEBVs for each trait, which was tested with a single marker regression as follows:

(2) $$y\;=\;\mu \;+X_{g}+e$$

where y is a vector of DEBVs for each trait (MY, FY, PY, and SCS); μ is overall mean; X is a design matrix allocating records to the marker effect; g is the effect of the SNP marker; and e is a vector of the random deviate $e_{ij}\sim N\left(0,\sigma_{e}^{2}\right)$, where $\sigma_{e}^{2}$ is the error variance. In this additive model, the marker effect is treated as a fixed effect (0, 1, and 2). The results were also clumped based on LD between SNPs using—clump flag in PLINK 1.9 with the default option (index variants were formed with *p*-values < 0.0001, and SNPs which are less than 250 kb away from an index variant and have *r*^2^ larger than 0.5 with it were removed). The significance threshold of the −log_10_ *p*-value ≥ 6.61 was determined based on Bonferroni correction. In addition, Quantile-Quantile (Q-Q) plots for each phenotype were performed to identify population stratification or cryptic relatedness.

#### 2.4.2. Bayesian C (BayesC) Approach

The general statistical model for the BayesC method was used with a π value of 0.997 and was fitted as follows:

(3) $$y\;=\;\mu \;+\overset{K}{\underset{k\;=\;1}{\sum }}Z_{k}\alpha_{k}+e$$

where *y* is an N×1 vector of DEBVs for each trait (MY, FY, PY, and SCS); μ is the overall mean; K is the number of SNP markers; Z is an N×1 vector of genotypes at SNP k; α is the additive effect of that SNP marker; and e is a vector of residual effects. In the present study, SNP genotypes were coded as the number of copies of one of the SNP alleles (i.e., −10, 0, and 10) using GenSel4R software [33]. The BayesC method assumes that SNP effects follow a normal distribution as a priori, and all SNPs have common variance. SNP marker effects were obtained using Gibbs sampling, and the initial 10,000 iterations out of a total of 110,000 Markov chain Monte Carlo (MCMC) iterations were excluded as part of the burn-in period with a thinning interval of five iterations. The significance level of the informative 1 Mb window region was 0.5% of the additive genetic variance, which was estimated as a portion of the total genetic variance explained by all SNPs.

### 2.5. Functional Annotations

The PPI analysis was performed based on each candidate gene with the 20 most interactive genes using the *Bos taurus* database and STRING v11.0 [34]. To investigate the functions of candidate genes, the Kyoto Encyclopedia of Genes and Genomes (KEGG) [35] pathway analysis was performed using the Database for Annotation, Visualization and Integrated Discovery (DAVID) v6.8 [36].

## 3. Results

### 3.1. General Statistics

Statistical information of phenotypes (DEBVs) in Korean Holstein is indicated in Table S4. The genotypes of the Korean Holstein population were analyzed using the imputed genotype data, and after QC, 201,704 out of 297,424 SNPs were used in the GWAS. For a total of 29 BTA autosomes, the average interval (± standard deviation) was 13,048.3 ± 14,905.6 bp. Among the 29 BTA autosomes after excluding sex chromosomes, BTA 8 had the largest number of SNPs (*n* = 15,260), and BTA 27 had the smallest number of SNPs (*n =* 2959). As for the average interval between SNPs, the longest and shortest were found on BTA 15 (16,344.2 bp) and BTA 23 (3881.9 bp), respectively. In the MDS plot, high similarities between individuals were identified (Figure S2).

### 3.2. GWAS Based on the SMR

**Figure 1:**
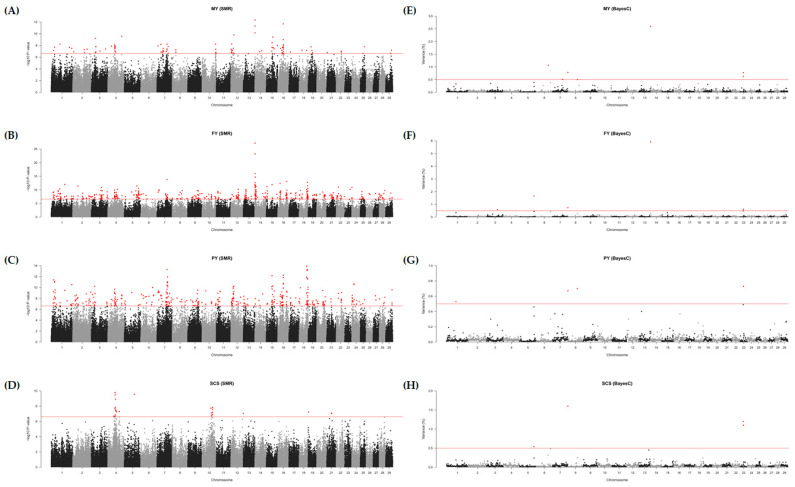
Manhattan plots for genome-wide association studies (GWAS) based on the single marker regression (SMR) and Bayesian C (BayesC) methods. GWAS performed in four phenotypes: (**A**,**E**) the adjusted 305-day milk yield (MY), (**B**,**F**) the adjusted 305-day fat yield (FY), (**C**,**G**) the adjusted 305-day protein yield (PY), and (**D**,**H**) the log_2_ transferred somatic cell score (SCS). Red dots represent significant SNPs or windows on SMR and BayesC, respectively.

For the four DEBV phenotypes (MY, FY, PY, and SCS) related to milk production, significant SNPs were identified based on the SMR determined by the Bonferroni correction (−log_10_ *p*-value ≥ 6.61) (Table S5 and Figure 1A–D). Q-Q plots for each phenotype indicated the possibility of a spurious association by population stratification or cryptic relatedness (Figure S3). The numbers of significant SNPs were 104, 491, 409, and 26 for MY, FY, PY, and SCS, respectively. For the MY phenotype, the most significant SNP was AX-311625843 (rs211223469, 1.7 Mb on BTA 14) with MAF of 0.266 located in the intron of *DGAT1*, and the highest SNP effect was observed in AX-115105679 (rs109608009, 39.0 Mb on BTA 16) with MAF of 0.015 located in the intron of paired related homeobox 1 (*PRRX1*). For the FY phenotype, the most significant SNP was AX-115099068 (rs109146371, 1.6 Mb on BTA 14) with MAF of 0.284 located in the upstream region of forkhead box H1 (*FOXH1*), and the highest SNP effect was observed in AX-320911501 (rs42774899, 46.8 Mb on BTA 17) with MAF of 0.016 located in the intergenic region. For the PY phenotype, the most significant SNP was AX-429486957 (rs383397306, 55.5 Mb on BTA 18) with MAF of 0.068 located in the intron of transmembrane protein 143 (*TMEM143*), and the highest SNP effect was observed in AX-429404258 (rs211516787, 45.0 Mb on BTA 19) with MAF of 0.010 located in the exon of the meiosis specific with coiled-coil domain (*MEIOC*). For the SCS phenotype, the most significant SNP was the AX-429899067 (rs210219823, 53.2 Mb on BTA 4) with MAF of 0.399 located in the intergenic region, and the highest SNP effect was observed in AX-310577007 (rs209990081, 35.9 Mb on BTA 21) with MAF of 0.053 located in the intergenic region. The abovementioned SNPs are marked in red in Table S5.

### 3.3. GWAS Based on the BayesC

To identify the significant 1-Mb windows including SNPs, GWAS were performed with four DEBV phenotypes (MY, FY, PY, and SCS) based on BayesC. We found 20 window regions including 40 informative SNPs based on their genetic effects (Table 1 and Figure 1E–H). For the MY phenotype, seven significant windows (≥0.5% genetic variance) were identified on BTA 14 (1 Mb), 6 (88 Mb), 8 (0 Mb), 23 (24 Mb), 23 (23 Mb), 7 (73 Mb), and 8 (69 Mb) (Figure 1E). Seven informative SNPs (AX-371638654, AX-311625833, AX-419656711, AX-311625845, AX-371657011, and AX-419792758) were identified in the window on BTA 14, which were annotated in heat shock transcription factor 1 (*HSF1*) and *DGAT1*. In addition, two SNPs (AX-185121607 and AX-106735408) were identified in the window on BTA 6, which were located in the intergenic regions. The remaining five windows presented one informative SNP each, indicating relatively low genetic effects. For the FY phenotype, five significant windows were identified on BTA 14 (1 Mb), 5 (104 Mb), 8 (0 Mb), 23 (24 Mb), and 3 (79 Mb) (Figure 1F). Thirteen informative SNPs (AX-429953677, AX-115099034, AX-371657011, AX-419793247, AX-419656711, AX-212342341, AX-419792758, AX-117081655, AX-124353826, AX-311625843, AX-311625845, AX-311625833, and AX-371638654) were identified in the window on BTA 14, which were annotated in spermatogenesis and centriole associated 1 (*SPATC1*), *DGAT1*, and *HSF1*. In addition, two SNPs (AX-106724308 and AX-169413290) were identified in the window on BTA 3, which were located in phosphodiesterase 4B (*PDE4B*). The remaining three windows presented one informative SNP each, indicating relatively low genetic effects. For the PY phenotype, four significant windows were identified on BTA 23 (24 Mb), 8 (69 Mb), 8 (0 Mb), and 1 (69 Mb) (Figure 1G), and for the SCS phenotype, four significant windows were identified on BTA 8 (0 Mb), 23 (23 Mb), 23 (24 Mb), and 5 (104 Mb) (Figure 1H). Those windows in the PY and SCS presented one informative SNP each, showing low genetic effects.

### 3.4. Fine Mapping and Candidate Genes

To identify the common significant regions, we compared the GWAS results between SMR and BayesC in each phenotype (MY, FY, PY, and SCS). For the MY phenotype, the common significant regions were the 1–2 Mb on BTA 14 and 73–74 Mb on BTA 7. It was noted that AX-311625843 (rs211223469) in *DGAT1* on BTA 14 showed significant effects in both methods (Figure S4A). For the FY phenotype, the common significant regions were the 1–2 Mb on BTA 14, 104–105 Mb on BTA 5, and 79–80 Mb on BTA 3 (Figure S4B). AX-419663582 (rs43454033) in *ANO2* on BTA 5 and AX-106724308 (rs42314807) in *PDE4B* on BTA 3 were shown to have significant effects in both methods. Although no common SNPs between SMR and BayesC were found on BTA 14, many significant and informative SNPs were found in each method. The other significant region was the 69–70 Mb on BTA 8 for the PY (Figure S4C). That region in PY was indicated to contain no common SNPs but was closely located. Contrary to the MY and FY phenotypes, the BayesC results of the PY and SCS showed that the informative SNPs had lower genetic effects (Table 1).

### 3.5. PPI and KEGG Pathway Analysis

**Figure 2:**
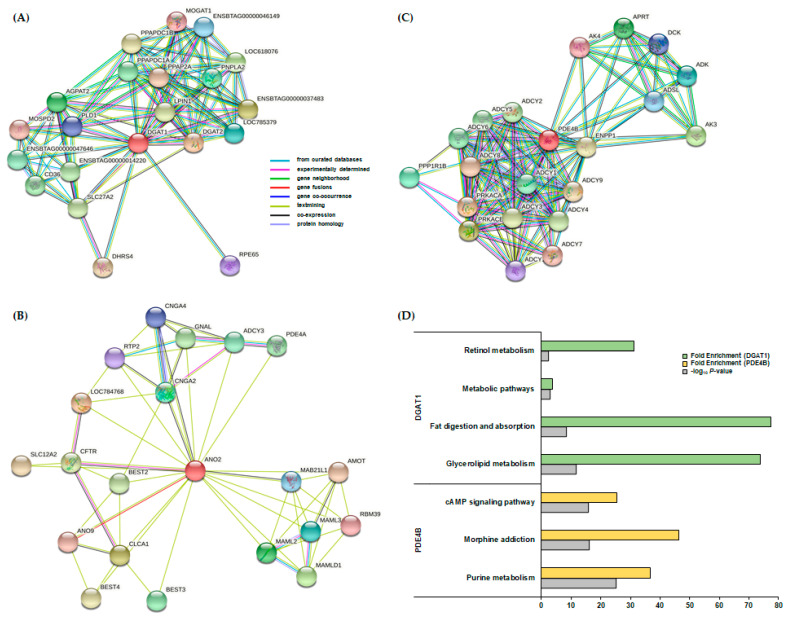
Protein–protein interaction (PPI) network and Kyoto Encyclopedia of Genes and Genomes (KEGG) pathway analyses for three candidate genes (*DGAT1*, *ANO2*, and *PDE4B*) with 20 interactor genes. (**A**) *DGAT1* with 20 interactors confirmed by PPI. (**B**) *ANO2* with 20 interactors were confirmed by PPI. (**C**) *PDE4B* with 20 interactors confirmed by PPI. (**D**) KEGG enrichment analysis for *DGAT1* and *PDE4B* with 20 interactors.

The three candidate genes (*DGAT1*, *ANO2*, and *PDE4B*) were selected based on GWAS using the two methods (SMR and BayesC) and were subjected to PPI identification (Figure 2A–C). Twenty interactors for each gene were determined in PPI, and a KEGG enrichment analysis was performed. *DGAT1*, the candidate gene for MY and FY, had interactor genes annotated in four significant KEGG pathways (*p*-value < 0.1): glycerolipid metabolism, fat digestion and absorption, metabolic pathways, and retinol metabolism (Figure 2D). *PDE4B*, the candidate gene for FY, had interactor genes annotated in three significant KEGG pathways: purine metabolism, morphine addiction, and cAMP signaling (Figure 2D). ANO2, the candidate gene for FY, had interactor genes that were not annotated in any significant KEGG pathways. Detailed information of the significantly enriched pathways is indicated in Table S6.

## 4. Discussion

We developed the Affymetrix Axiom Bovine Custom300K customized chip for dairy cattle to utilize fine mapping methods and performed GWAS for four milk production phenotypes (MY, FY, PY, and SCS) in a Korean Holstein population. The associations between SNPs and phenotypes were analyzed based on two methods (SMR and BayesC). Many previous studies reported comparisons between these two methods [37,38,39,40,41].

The SMR has been used in many studies to address data from GWAS. The SMR and its modified methods were developed to detect the large effects of each SNP, but they are likely to overestimate the genetic effect of SNPs because they ignore the effects of other SNPs [42]. Although the Q-Q plots showed inflations by population stratification or cryptic relatedness (Figure S3), it can be postulated by features of the Axiom Bovine Custom300K involving high-density SNPs in QTL regions such as *DGAT1* that MDS indicated high similarities in this population (Figure S2). The BayesC method that estimates all feasible genetic effects has been used as an alternative or improved method for QTL mapping or GWAS [42,43,44]. Although both SMR and BayesC methods are effective for detecting markers with large effects, the latter is more effective for detecting markers without overestimation [45,46]. BayesC with a high π value tends to highlight SNPs with large effects (variance), as they assume the prior distribution of the selected SNP effects (variances), suggesting that the SNP effect may be detected more clearly in the BayesC method than in the SMR [43]. Considering that these two methods have their own strengths and weakness, the present study focused on common regions and SNPs detected by both methods.

Five regions were identified to have significant effects in both methods in the GWAS and fine mapping of four phenotypes (MY, FY, PY, and SCS) (Figure S3). The BTA 14 (1–2 Mb) region that was found to be significant in both MY and FY has been reported to be significant in Chinese and U.S. Holstein populations [14,47]. In addition, BTA 7 (73–74 Mb) for MY and BTA 5 (104–105 Mb) for FY have also been reported to be significant in a Chinese Holstein population [11,48]. However, two remaining regions (BTA 3: 79–80 Mb and BTA 8: 69–70 Mb) were first reported in the present study.

AX-311625843 (rs211223469) at the 1–2 Mb in *DGAT1* on BTA 14, in particular, is one of the most important SNPs, as it affects both MY and FY phenotypes. It was suggested that BTA 14 is an important QTL region [49,50,51] and that the *DGAT1* gene is a causal gene related to the QTL region [52,53,54]. The *DGAT1* gene produces an enzyme that catalyzes the final step in synthesizing triglyceride, which makes up about 98% of milk fat [55,56], and plays an important role in synthesizing triglyceride in mammary glands [57,58]. In addition, the *DGAT1* gene encodes a key metabolic enzyme catalyzing the biosynthesis of triacylglycerols and participates in glycerolipid metabolism, retinol metabolism, and fat digestion and absorption as indicated by the KEGG pathway analysis and as reported previously [11,59]. The *DGAT1* gene is considered to affect FY as well as MY in dairy cattle through physiological mechanisms.

AX-106724308 (rs42314807) at the 79–80 Mb in *PDE4B* on BTA 3 was identified to have a significant effect on FY in both methods. Previous studies observed a significantly strong relationship between the *PDE4B* gene and milk-related traits (MY, FY, and PY) [60,61,62]. PDE4 is cAMP-specific, known to be inhibited by rolipram [63,64], and is the biggest PDE family encoded by four genes (*PDE4A*, *PDE4B*, *PDE4C*, and *PDE4D*), which has been reported to be involved in various functional activities such as cell desensitization or adaptation, signaling cross-talk and cAMP signal compartmentalization. Although the correlation between the level of PDE4 and cAMP concentration remains unclear, this family is considered to be an important cAMP homeostatic regulator [65]. PDE4 transcripts and proteins were previously detected in cattle mammary glands, as were active enzymes, suggesting a functional role [66]. There were three significant KEGG pathways (purine metabolism, morphine addiction, and cAMP signaling pathway) based on the *PDE4B* with 20 interactor genes revealed by the KEGG enrichment analysis. Among the significant KEGG pathways, the cAMP signaling pathway was related to FY through the regulation of AMP-activated protein kinases by cAMP in adipocytes. This physiological mechanism was reported in previous studies [66,67,68].

AX-419663582 (rs43454033) at the 104–105 Mb in *ANO2* on BTA 5 was identified to have a significant effect on FY in both methods. In previous studies, this region of BTA 5 was shown to have many QTL related to milk fat content, a characteristic of milk production [9,54,62,69]. The functions of the *ANO2* gene in this region, however, were not reported in relation to milk content.

The region identified by both methods to have a significant effect on PY was the 69–70 Mb region on BTA 8, which was also shown to be a QTL region related to PY in previous studies [11,70]. The results of BayesC showed that, among the 2,288 SNPs across 1 Mb, there were almost no differences between the largest marker effect and the remaining ones. Therefore, these results indicate that the PY phenotype has a lower genetic effect than the MY and FY phenotypes.

As milk is produced through mammary glands, the health status of these glands is especially important, and the SCS is used as an index of health status. The somatic cells refer to the leukocytes of milk in cows. For example, as the leukocytes around the mammary glands of a dairy cow suffering from mastitis increase rapidly, and these leukocytes are reflected in the SCS of milk, this score indicates the presence of infection and the health status of the cow. A lower SCS represents a healthier cow as well as a higher quality of milk [71,72,73,74,75]. QTLs related to SCS were previously detected on BTA 5, 6, 8, 11, 17, 18, 20, and 23, and we detected them on BTA 8 (0–1 Mb), BTA 23 (23–25 Mb), and BTA 5 (104–105 Mb) associated with the SCS phenotype [76,77,78]. Similar to the results for the PY phenotype, the BayesC results showed no specific SNPs with significantly high genetic effects which were shared without noticeable difference. Therefore, we recommend that additional fine mapping should be performed, and the candidate gene approach should be more precisely applied to detect causal genes of PY and SCS phenotypes in Korean Holstein.

## 5. Conclusions

A GWAS was performed to analyze milk production phenotypes (MY, FY, PY, and SCS) in a Korean Holstein population, and six QTL regions (MY: 1–2 Mb on BTA 14 and 73–74 Mb on BTA 7; FY: 1–2 Mb on BTA 14, 104–105 Mb on BTA 5, and 79–80 Mb on BTA 3; PY: 69–70 Mb on BTA 8) were detected. In addition, *DGAT1*, *ANO2*, and *PDE4B* were subjected to PPI network and KEGG enrichment analyses, and the results showed that the *DGAT1* gene is involved in glycerolipid metabolism, fat digestion and absorption, metabolic pathways, and retinol metabolism pathways, affecting the MY and FY phenotypes. The *PDE4B* gene was found to be closely involved in metabolic activity through the cAMP signaling pathway, affecting the FY phenotype. No strong candidate genes, however, were selected for the PY and SCS phenotypes, though significant regions were identified, suggesting the necessity of further studies. Our findings are expected to provide important information for the genomic selection of those phenotypes to improve milk production in Korean Holstein cattle.

## Figures and Tables

**Table 1:** Summary of informative SNPs in the significant 1-Mb windows associated with DEBV phenotypes (MY, FY, PY, and SCS) based on BayesC.

PHENOTYPE (DEBV) ^a^	BTA (Mb) ^b^	GV (%) ^c^	Informative SNP	Rs Number	Position ^d^	SNP Effect	Model Frequency	Region Annotation	Gene Annotation
MY	14 (1)	2.60	AX-371638654	rs211016627	1,807,655	23.9100	0.1845	Intron	*HSF1*
			AX-311625833	rs384957047	1,793,616	−22.1900	0.1717	Upstream gene	*DGAT1* (dist = 1735)
			AX-311625843	rs211223469	1,799,476	−20.4900	0.1617	Intron	*DGAT1*
			AX-419656711	rs211282745	1,805,963	−17.5500	0.1415	Downstream gene	*HSF1* (dist = 118)
			AX-311625845	rs209876151	1,800,439	−15.9000	0.1300	Intron	*DGAT1*
			AX-371657011	rs208640292	1,806,875	12.8000	0.1095	Synonymous	*HSF1*
			AX-419792758	rs207655744	1,806,340	12.3300	0.1057	3 prime UTR	*HSF1*
	6 (88)	1.03	AX-185121607	rs110775601	88,952,089	78.4400	0.6617	Intergenic	*NPFFR2* (dist = 100,121)
			AX-106735408	rs110527224	88,592,295	17.9900	0.1741	Intergenic	*SLC4A4* (dist = 54,249)
	8 (0)	0.78	AX-419764649	rs721532493	887,406	−1.0640	0.0183	Intron	*PALLD*
	23 (24)	0.77	AX-419655926	rs380223715	24,021,950	−1.3030	0.0180	Intron	*PKHD1*
	23 (23)	0.63	AX-419634159	rs517703887	23,999,941	−1.0560	0.0169	Intron	*PKHD1*
	7 (73)	0.51	AX-169404932	rs135477609	73,561,312	4.0460	0.0477	Intergenic	*ADRA1B* (dist = 49,805)
	8 (69)	0.50	AX-419751453	rs524049037	69,514,127	−1.7210	0.0251	Intron	*GFRA2*
FY	14 (1)	5.92	AX-429953677	rs110812136	1,991,225	1.9290	0.3670	Intron	*SPATC1*
			AX-115099034	rs109421300	1,801,116	1.5190	0.2793	Intron	*DGAT1*
			AX-371657011	rs208640292	1,806,875	−1.1010	0.2097	Synonymous	*HSF1*
			AX-419793247	rs208317364	1,800,399	−0.9766	0.1922	Intron	*DGAT1*
			AX-419656711	rs211282745	1,805,963	0.8508	0.1682	Downstream gene	*HSF1* (dist = 118)
			AX-212342341	rs135258919	1,808,145	0.8499	0.1693	Missense	*HSF1*
			AX-419792758	rs207655744	1,806,340	−0.8430	0.1675	3 prime UTR	*HSF1*
			AX-117081655	rs109234250	1,802,265	−0.7752	0.1565	Missense	*DGAT1*
			AX-124353826	rs109326954	1,802,266	−0.6848	0.1395	Missense	*DGAT1*
			AX-311625843	rs211223469	1,799,476	0.6695	0.1367	Intron	*DGAT1*
			AX-311625845	rs209876151	1,800,439	0.6313	0.1319	Intron	*DGAT1*
			AX-311625833	rs384957047	1,793,616	0.5941	0.1243	Upstream gene	*DGAT1* (dist = 1735)
			AX-371638654	rs211016627	1,807,655	−0.4948	0.1052	Intron	*HSF1*
	5 (104)	1.66	AX-419663582	rs43454033	104,831,727	−0.1767	0.0560	Intron	*ANO2*
	8 (0)	0.74	AX-419764649	rs721532493	887,406	−0.0319	0.0143	Intron	*PALLD*
	23 (24)	0.62	AX-419669189	rs435871639	24,210,330	−0.0168	0.0086	Intron	*PKHD1*
	3 (79)	0.60	AX-106724308	rs42314807	79,480,234	−1.8050	0.4151	Intron	*PDE4B*
			AX-169413290	rs41596885	79,508,402	0.7012	0.1766	Intron	*PDE4B*
PY	23 (24)	0.73	AX-419655926	rs380223715	24,021,950	−0.0118	0.0086	Intron	*PKHD1*
	8 (69)	0.70	AX-419606850	rs211419403	69,542,993	−0.0259	0.0144	Intron	*GFRA2*
	8 (0)	0.67	AX-419764649	rs721532493	887,406	−0.0214	0.0137	Intron	*PALLD*
	1 (69)	0.53	AX-419771850	rs799074643	69,736,662	0.0607	0.0294	Intron	*UMPS*
SCS	8 (0)	1.60	AX-419631051	rs109008410	668,048	0.0012	0.0253	Intron	*PALLD*
	23 (23)	1.20	AX-312701115	rs467721520	23,807,184	−0.0006	0.0150	Intron	*PKHD1*
	23 (24)	1.10	AX-106721976	rs109825181	24,117,682	−0.0012	0.0246	Intron	*PKHD1*
	5 (104)	0.54	AX-124344695	rs110571898	104,682,238	0.0005	0.0127	Missense	*VWF*

^a^ MY: Adjusted-305 days milk yield (kg); FY: Adjusted-305 days fat yield (kg); PY: Adjusted-305 days protein yield (kg); SCS: log_2_ transferred somatic cell score; ^b^ *Bos taurus* autosome; ^c^ Genetic variance; ^d^ The positions were based on UMD3.1.

## Supplementary Materials

The following are available online at https://www.mdpi.com/article/10.3390/ani11051392/s1, Figure S1: The distribution for reliabilities of EBVs in each phenotype: (A) MY, (B) FY, (C) PY, and (D) SCS; Figure S2: Multidimensional scaling (MDS) plot based on SNPs for Korean Holstein population; Figure S3: Quantile-Quantile (Q-Q) plots for 4 milk production phenotypes based on single marker regression (SMR). (A) Q-Q plot for MY. (B) Q-Q plot for FY. (C) Q-Q plot for PY. (D) Q-Q plot for SCS; Figure S4: Visualization of the fine mapping in common significant regions by genome-wide association studies (GWAS) based on single marker regression (SMR) and Bayesian C (BayesC). (A) Common significant regions for MY. (B) Common significant regions for FY. (C) Common significant region for PY. Left Y- and Right Y-axis present effect (β, blue) in SMR and 1-Mb window genetic variance (red) in BayesC, respectively. Genes included in 1-Mb windows were annotated; Table S1: Variance components and heritability for four milk production phenotypes in Korean Holstein (*n* = 2780); Table S2: Summary of genotyped animals in each SNP genotyping platform; Table S3: Information of total SNPs in the Affymetrix Axiom Bovine Custom300K chip platform; Table S4: General statistics of DEBVs for 4 milk production phenotypes in Korean Holstein; Table S5: Summary of significant SNPs associated with DEBV phenotypes (MY, FY, PY, and SCS) based on SMR; Table S6: Significantly enriched KEGG pathways based on *DGAT1* and *PDE4B* with 20 interactors.
