# Supplementary material for: Genome-Wide Identification of Candidate Genes for Milk Production Traits in Korean Holstein Cattle
Source: Animals (Basel). 2021 May 13;11(5):1392. doi: 10.3390/ani11051392 (PMC8153329; doi:10.3390/ani11051392)
Supplement: Supplementary file 1 [file animals-11-01392-s001.zip › animals-1192801-supplementary material/animals-1192801-supplementary material.pdf]

animals

Genome-Wide Identification of Candidate Genes for Milk Production Traits in Korean Holstein Cattle

Sangwook Kim [1,†], Byeonghwi Lim [1,†], Joohyeon Cho [2], Seokhyun Lee [2], Chang-Gwon Dang [3], Jung-Hwan Jeon [4], Jun-Mo Kim [1,*] and Jungjae Lee [1,*]

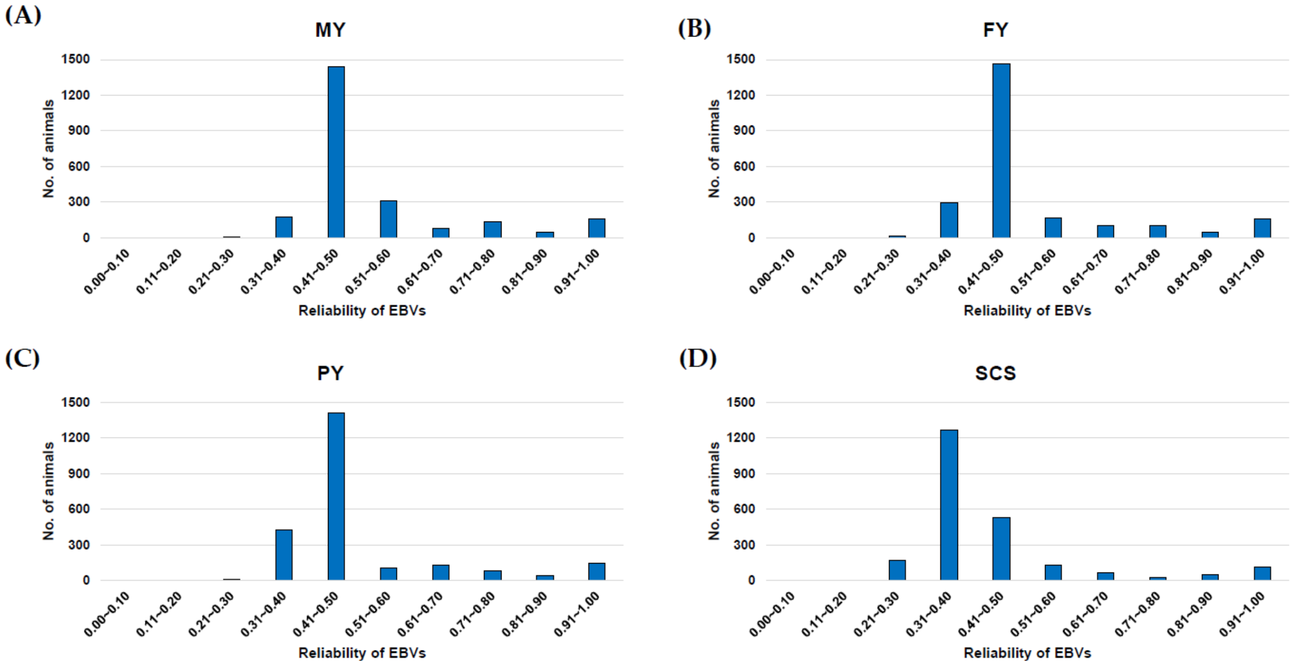

Figure S1. The distribution for reliabilities of EBVs in each phenotype: (**A**) MY, (**B**) FY, (**C**) PY, and (**D**) SCS.

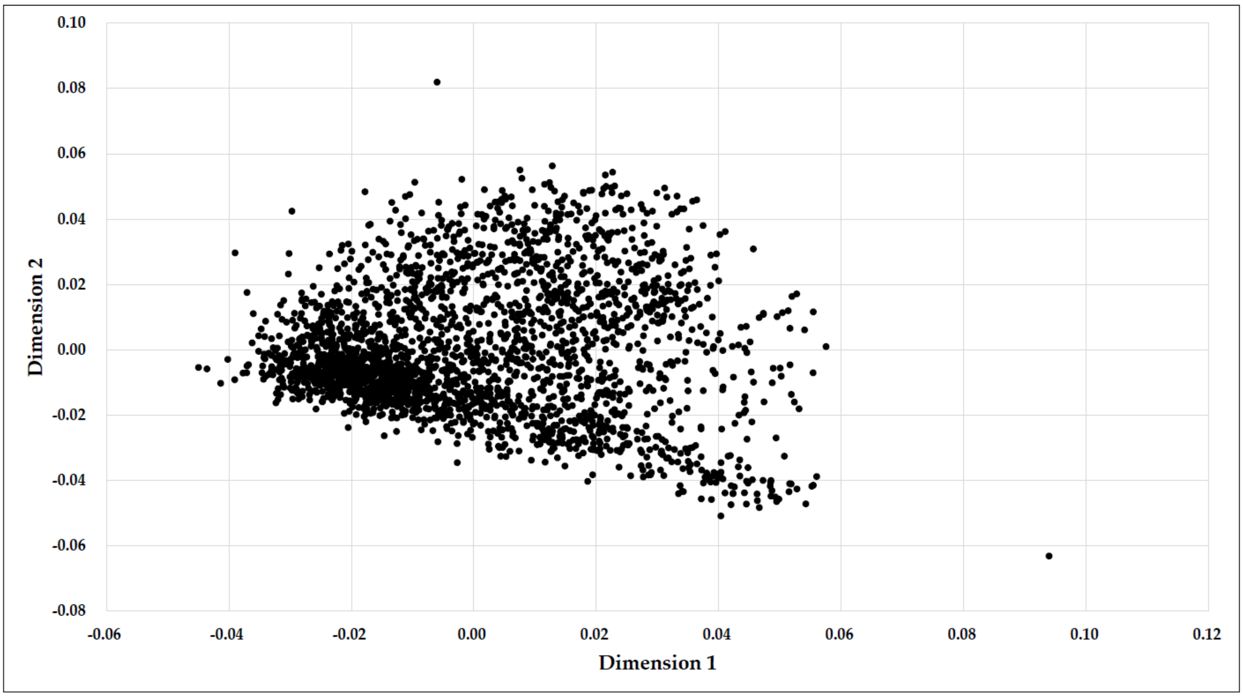

Figure S2. Multidimensional scaling (MDS) plot based on SNPs for Korean Holstein population.

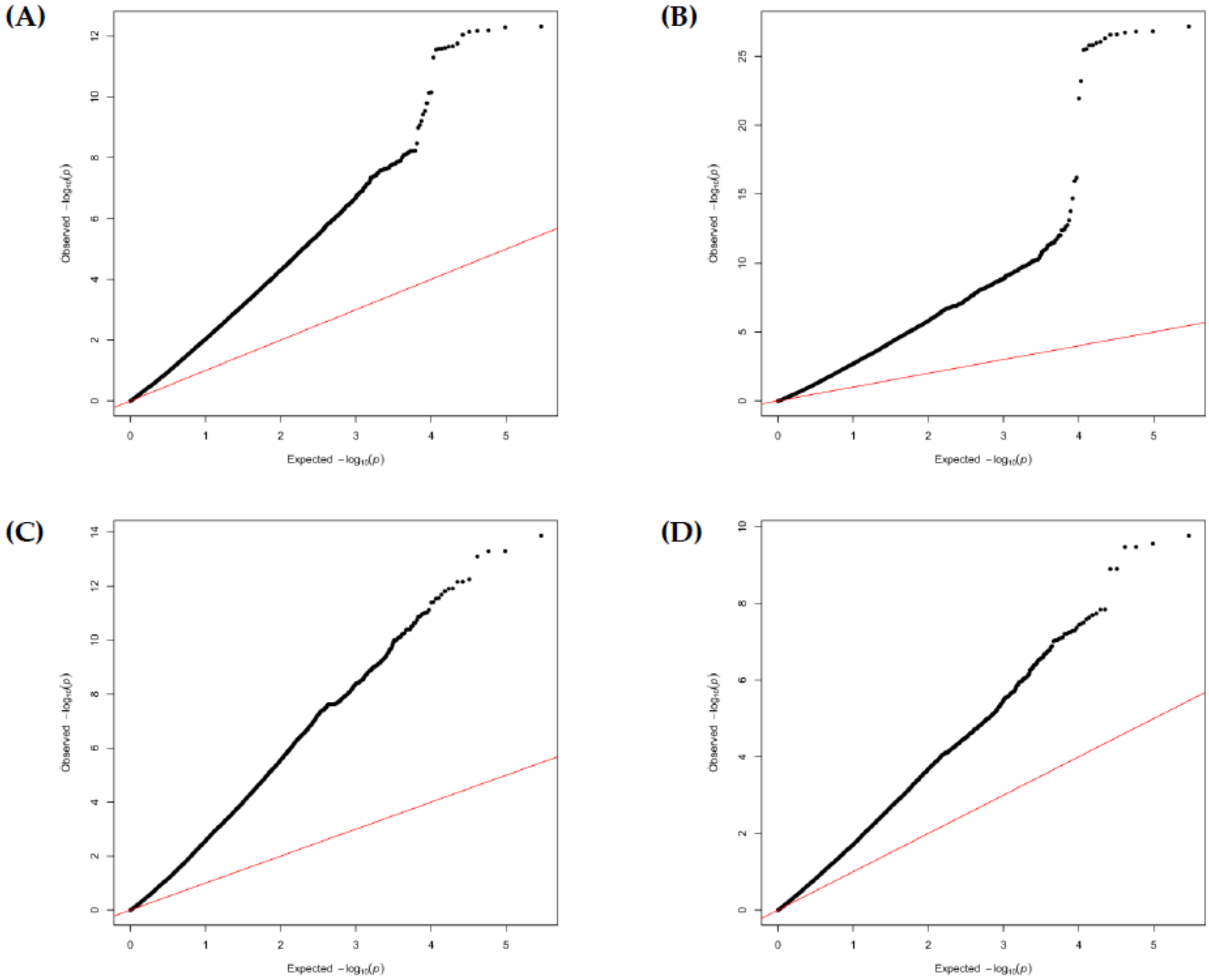

Figure S3. Quantile-Quantile (Q-Q) plots for 4 milk production phenotypes based on single marker regression (SMR). (**A**) Q-Q plot for MY. (**B**) Q-Q plot for FY. (**C**) Q-Q plot for PY. (**D**) Q-Q plot for SCS.

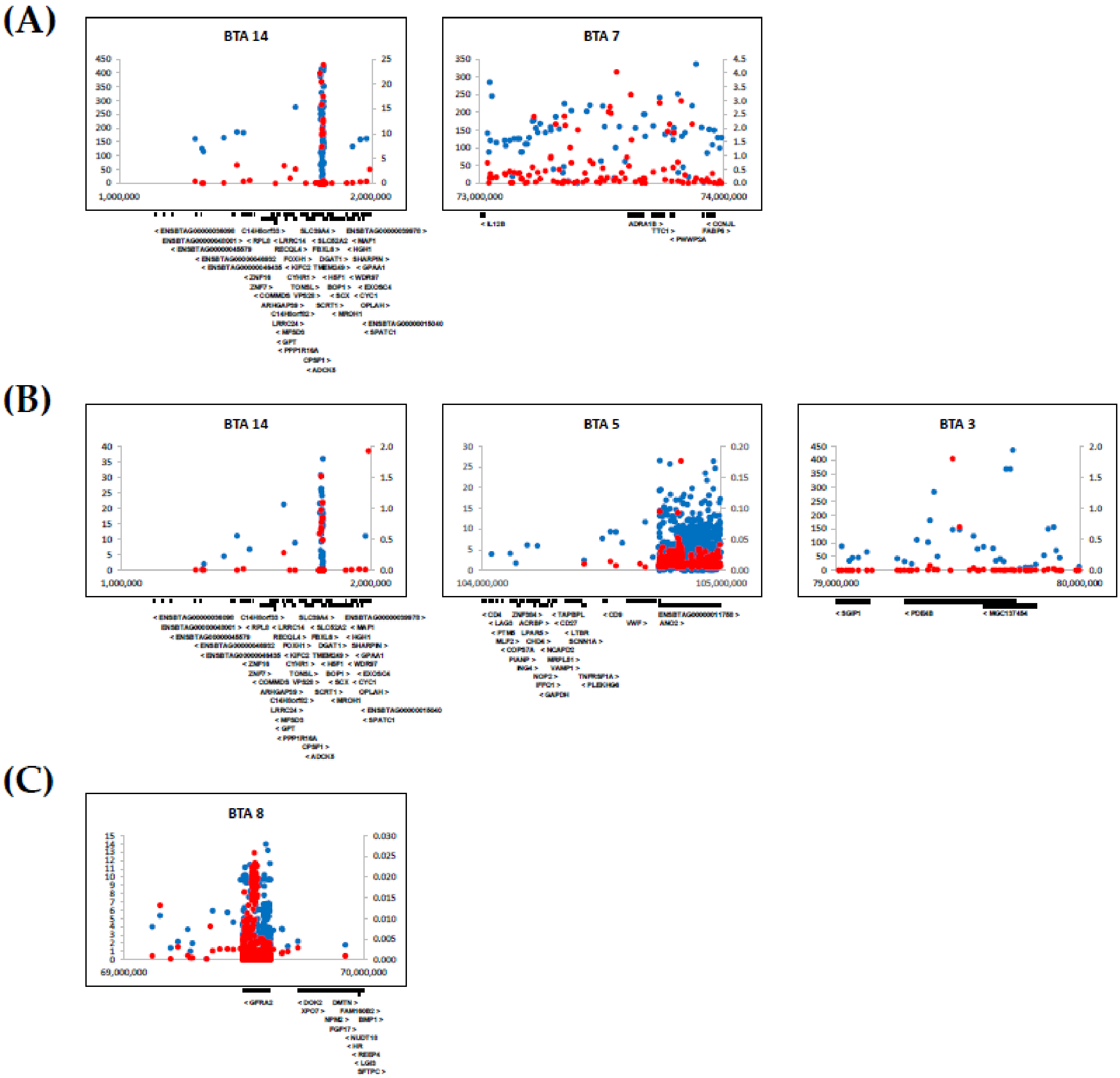

Figure S4. Visualization of the fine mapping in common significant regions by genome-wide association studies (GWAS) based on single marker regression (SMR) and Bayesian C (BayesC). (**A**) Common significant regions for MY. (**B**) Common significant regions for FY. (**C**) Common significant region for PY. Left Y- and Right Y-axis present effect (β, blue) in SMR and 1-Mb window genetic variance (red) in BayesC, respectively. Genes included in 1-Mb windows were annotated.
